# Metagenomic Insights into the Microbiome and Resistance Genes of Traditional Fermented Foods in Arabia

**DOI:** 10.3390/foods12183342

**Published:** 2023-09-06

**Authors:** Muhammad Yasir, Areej A. Alkhaldy, Samah Abdullah Soliman, Safaa A. Turkistani, Esam I. Azhar

**Affiliations:** 1 Special Infectious Agents Unit, King Fahd Medical Research Center, King Abdulaziz University, Jeddah 21589, Saudi Arabia; 2 Department of Medical Laboratory Sciences, Faculty of Applied Medical Sciences, King Abdulaziz University, Jeddah 21589, Saudi Arabia; 3 Clinical Nutrition Department, Faculty of Applied Medical Sciences, King Abdulaziz University, Jeddah 21589, Saudi Arabia; 4 Department of Nursing, Dr. Soliman Fakeeh Hospital, Jeddah 21134, Saudi Arabia; 5 Fakeeh College for Medical Sciences, Jeddah 21134, Saudi Arabia

**Keywords:** fermented foods, mish, dairy fermented food, bacterial communities, metagenomic, antimicrobial resistance, Saudi Arabia

## Abstract

This study uncovered microbial communities and evaluated the microbiological safety of traditional fermented foods consumed in the Arab region. Samples of dairy and non-dairy fermented foods—mish, jibneh, zabadi, and pickles—were collected from local markets in Saudi Arabia. Using the MiSeq system, samples were sequenced using 16S amplicons and shotgun metagenomics. Alpha and beta diversity indicated inter- and intra-variation in the studied fermented foods’ bacterial communities. In the case of mish, the replicates were clustered. Twenty-one genera were found to be significantly different (FDR < 0.05) in abundance in pairwise comparison of fermented foods. Five high-quality, metagenome-assembled genomes (MAGs) of *Lactococcus lactis*, *Lactobacillus helveticus*, *Pseudoalteromonas nigrifaciens*, *Streptococcus thermophiles*, and *Lactobacillus acetotolerans* were retrieved from the shotgun sequencing representing the dominant taxa in the studied fermented foods. Additionally, 33 genes that cause antimicrobial resistance (ARGs) against ten different antibiotic classes were detected. Metabolic pathways were abundant in the studied metagenomes, such as amino acid metabolism, carbohydrate metabolism, cofactors, and vitamin biosynthesis. Metagenomic evaluation of Arabian fermented foods, including the identification of probiotics, pathogenic bacteria, and ARGs, illustrates the importance of microbiological analysis in evaluating their health effects.

## 1. Introduction

Saudi Arabia occupies most of the Arabian Peninsula, and most of its population descended from nomadic tribes who historically lived throughout the region. Saudi Arabian society has rapidly shifted from nomadic to urban life in the last fifty years. Despite the modern advancements that have come with this transformation, many people continue to raise goat, sheep, and camel herds. Their daily meals consist of fermented milk products such as laban, mish, cheese, and yogurt, as well as non-dairy, traditional vegetable pickles [1,2]. Although the health benefits of fermented foods have been well known to the inhabitants of the Arabian Peninsula since ancient times, the indigenous fermented foods of Saudi Arabia have not been thoroughly studied in terms of microbial community composition, antibiotic resistance profile, and safety assessment [3].

Fermentation of foods is an outcome of metabolic activities of complex and diverse microbial floras, which multiply using the natural ingredients of the food and are transferred to a wide range of molecules that provide a distinctive composition to the final fermented products [4,5]. The diversity and community profile of such fermented food microbiota is specific for each food and depends on the ingredients used for fermentation and the production process [2,6,7]. The dairy and agro-food industries use commercial mixtures of specific strains for fermentation, and the microbiological profile of those products is more standardized [8]. Traditional fermentation is probably the result of bacterial strains originating from the environment, particularly in raw ingredients that have been fermented [6,9]. Several studies, mainly from China and other Western countries, have reported the microbiota and functional metagenome of different fermented food, including rubing milk cake, cheese, yan-cai pickle, nunu (Ghanaian fermented milk), suero costeno, fermented liquor, and fermented sausages [9,10,11]. Fermented foods typically contain beneficial probiotic bacteria, such as *Lactobacillus delbrueckii* and *Streptococcus thermophilus*, but may also harbor harmful pathogens, including *Salmonella*, *Listeria monocytogenes*, and *Staphylococcus aureus* [2,4,11].

The significance of the food microbial community for human health becomes apparent by considering its impact on the human gut’s microbiome composition and colonization [12,13,14]. The microbiome of the human digestive tract comprises a complex and heterogeneous community of 100 trillion microbial cells classified in over 1000 species, many of which stay with their hosts in symbiotic relationships [14]. Gut microbiota play an important role in nutrient processing, host energy metabolism, maintenance of immune homeostasis, and protection against pathogens [14,15,16]. An increasing number of studies have marked the role of food in shaping the composition of gut flora, particularly of Firmicutes and Bacteroidetes composition, the two dominant phyla residing in the gastrointestinal tract [12,17]. For instance, the consumption of probiotics in large quantities could influence the composition of gut microbiota through the production of antimicrobial molecules, such as bacteriocins and short chain fatty acids, inhibiting colonization of pathogens through mucin production, and potentially enhancing the growth of specific groups of microbial species [17,18,19].

A variety of fermented foods are famous in Saudi Arabia, some of which are prepared through specific local recipes and processes, such as traditional fermented sameel, mish, laban, gariss, zabadi (yogurt), jibneh (cheese), and mixed vegetable pickles. Fermented dairy products can be divided into two main categories: the truly indigenous, such as roub, gariss, and mish, and the quasi-indigenous, such as zabadi and jibneh [20]. Traditionally, mish fermented milk is a spiced blend of cumin (*Nigella sativa*), fenugreek (*Trigonella foenum-graecum*), and occasionally garlic (*Allium sativa* L.) [21]. In the mish, spice intensity may differ from one region to another, depending on spice availability and particular taste preferences [21]. The microbiota of these local fermented foods have rarely been studied by culturing method. The characterization of microbiota of traditionally fermented foods is not only critical from their prospective role in the development of gut microbiome but it is also important from the food safety point of view [22].

The microbial flora found in traditionally fermented foods comes from the environment, and antibiotic resistance in food-borne bacteria has recently received attention [2,6]. Antimicrobial resistant genes (ARGs) content can grow in fermented foods due to bacterial multiplication and horizontal gene transfer since fermented foods contain high titers of live bacteria. Leech et al., observed differences in fermented foods’ resistome according to food type and fermentation method in a metagenomic study [6]. Multidrug resistance genes were commonly found among these foods, followed by beta-lactam resistance genes were common in dairy and sugar foods [6]. Brine-type food contained tetracycline resistance genes [6]. From pickle samples, 285 ARGs and variants were identified from shotgun metagenomic in a previous study [7]. The antibiotic resistant strains and resistance genes pool in fermented products increased the risk of ARGs transfer to human pathogens which might be present within the gut, reviewed in detail previously [23]. Food products in Europe are regulated for safety by the European Food Safety Authority (EFSA). The World Health Organization has developed guidelines to assist stakeholders in establishing integrated antimicrobial resistance surveillance in food-borne bacteria. It is recommended to frequently check the microbiological safety of indigenous fermented foods sold in the local markets of Saudi Arabia. The aim of this study was to characterize microbiota and evaluate safety by identifying pathogenic bacteria and ARGs in indigenous Arabian dairy fermented foods, mish, zabadi, jibneh, and mixed vegetable pickles using a next generation sequencing based metagenomic approach.

## 2. Materials and Methods

A total of nine samples from three different dairy fermented foods and two samples from non-dairy mixed vegetable pickles were collected from local markets in Saudi Arabia. Replicate biological samples of each fermented food [mish (MIS), jibneh (JIB), zabadi (ZAB), and pickle (PIC)] were collected aseptically in sterilized containers. The samples were kept in an icebox during transportation and then stored at a temperature of −80 °C in the laboratory.

### 2.1. 16S Amplicon Sequencing and Bioinformatics Analysis

Briefly, to remove the fat layer from dairy foods, 500 mg of each sample was resuspended in 1.5 mL of phosphate-buffered saline and vortexed for 30 s. The tubes were centrifuged for 10 min at 13,000× *g*. The top fatty layer and supernatant were discarded, and the pellet was used for DNA extraction with the beads-beating method of DNeasy PowerSoil Pro Kit (Qiagen, Berlin, Germany). The beads-beating method is efficient at extracting DNA from a wide variety of microorganisms, and the selected kit also removes PCR inhibitors. DNA extraction from pickle samples was performed following the removal of the vegetable debris using cheesecloth as described previously [7]. The concentration of DNA was measured using the Qubit Fluorometer and the Qubit dsDNA high-sensitivity kit (Invitrogen, Waltham, MA, USA). The 16S amplicon sequencing was performed with the use of universal primers 341F and 785R, designed to target the V3-V4 region of the gene. Illumina’s MiSeq system (Illumina, Inc., San Diego, CA, USA) was used to conduct next generation sequencing, utilizing a 2 × 300 bp paired end chemistry of a 600 cycle kit, as previously described [24]. 16S amplicon sequencing was classified through the BaseSpace 16S Metagenomics application v1.1.0 (Illumina, Inc.) that used the GreenGenes SQL taxonomic database curated by Illumina and RefSeq RDP 16S v3 database with the ribosomal database project classifier algorithm [25]. The Kolmogorov–Smirnov D test was applied to check the data normality. Alpha and beta diversity analysis was performed using the MicrobiomeAnalyst pipeline 2.0 [26]. The significance of beta diversity among the studied fermented foods was evaluated with principal coordinate analysis (PCoA) based on permutational MANOVA.

### 2.2. Shotgun Sequencing and Bioinformatics Analyses

One sample from each fermented food was processed for shotgun sequencing. DNA libraries were prepared with insert sizes of approximately 400 bp using the Nextera XT DNA Library Preparation Kit (Illumina, Inc.). The libraries were quantified, and quality control was made using Agilent D1000 HS tapes on the TapeStation 4200 (Agilent Technologies, Santa Clara, CA, USA). Libraries were pooled at equimolar amounts, and sequencing was processed with 2 × 300 bp chemistry on a MiSeq instrument (Illumina, Inc.) using 600-cycle kit (Illumina, Inc.,). Metagenomic analysis was performed using the Metagenomic Rapid Annotations using Subsystems Technology (MG-RAST) and the KBase platform [27,28].

The microbial community was elucidated from unassembled sequence reads using the Kaiju program [29]. Trimmomatic v0.36 was used to trim barcodes [30]. MaxBin2 v2.2.4 was used to reconstruct representative metagenome-assembled genomes (MAGs) from genomic assemblies prepared with metaSPAdes v3.15.3. The percentages of completion and contamination of the MAGs were examined using the CheckM version 1.0.18. After extracting MAGs from binned contigs through a tool of Extract Bins as Assemblies from Binned Contigs app v1.0.2, they were annotated using RAST (Rapid Annotations using Subsystems Technology). MAGs were analyzed using GTDB-Tk programs for their taxonomy. A maximum-likelihood phylogenetic tree was constructed with the Insert Set of Genomes into Species Tree v2.1.10 program using MAGs with closely related genomes from the NCBI microbial genome database. The interactive Tree of Life tool was used to visualize the phylogenetic tree.

To conduct functional analysis, we mapped the open reading frames to the Kyoto Encyclopedia of Genes and Genomes (KEGG) orthology (KO) databases using the MG-RAST platform with default settings [31]. ARGs were identified from the short sequence reads with CZ ID, a web-based platform for RGI analysis of metagenomic sequencing reads using the Comprehensive Antibiotic Resistance Database (CARD 3.1.4) [32]. Relative abundance was used for taxonomic and functional analysis of the metagenomic data. ARGs were normalized to reads per million (rpm). Standard deviations were calculated with SPSS v22 software. Venn diagrams were constructed using the InteractiVenn tool.

## 3. Results

### 3.1. 16S Amplicon Sequencing Taxonomic Analysis

**Figure 1:**
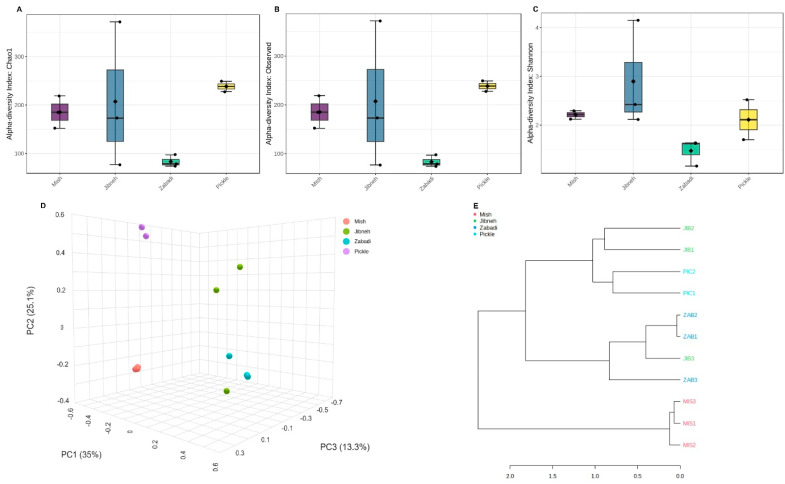
Alpha and beta diversity analysis of dairy and non-dairy fermented foods using 16S amplicon sequencing. Alpha diversity analysis: (**A**) chao1, (**B**) observed species, and (**C**) Shannon diversity index. Beta diversity analysis: (**D**) principal coordinate analysis revealed variation in bacterial communities across samples, and (**E**) hierarchical clustering using Bray–Curtis dissimilarity analysis.

Based on the 16S amplicon, 0.37 million high-quality sequence reads were obtained from the fermented food samples. Sequence reads were classified into 844 OTUs at the species taxonomic level with 97% similarity and assigned to microbial domains. In alpha diversity, a high level of divergence in bacterial diversity was apparent in the jibneh samples compared to other fermented foods (Figure 1A–C). According to the Shannon diversity index analysis, the jibneh had an increased diversity of bacteria followed by pickle and mish, whereas the least bacterial diversity was found in zabadi (Figure 1C). Overall, differences across fermented food samples, according to the chao1 values (*p* = 0.24), observed species (*p* = 0.24), and Shannon diversity index (*p* = 0.15) were statistically not significant. PCoA analysis revealed separate clustering of each fermented food that was statistically significant (*p* = 0.01). The intra-replicates variation was more apparent in jibneh samples, whereas mish replicates were clustered (Figure 1D). Bray–Curtis dissimilarity analysis identified separate hierarchical clustering of mish samples from other fermented foods, whereas the pickle samples clustered with jibneh samples (Figure 1E).

The 16S amplicon sequencing reads were assigned to 22 bacterial phyla, where 15 phyla were commonly found in >50% of the fermented foods samples. Firmicutes was the only dominant phylum in most of the studied samples and was detected at >90% relative abundance in zabadi samples (Figure S1). The traditional Arabian mish samples carried a high abundance of both Firmicutes (53.2% ± 6.2%) and Proteobacteria (46.3% ± 6.1%). Among other phyla, Bacteroidetes, Actinobacteria, and Verrucomicrobia were commonly found in all studied samples, but relative abundance of the common phyla varied in the dairy and non-diary fermented foods (Figure S1). Bacilli from Firmicutes were predominantly found in mish (52% ± 6.7%) and other dairy and pickle samples, whereas Gammaproteobacteria were primarily found in the mish (45.9% ± 6%), two of the jibneh (JIB1 = 67.4%, JIB2 = 46.9%) samples and a mixed vegetable pickle (PIC2 = 10.8%) sample.

**Figure 2:**
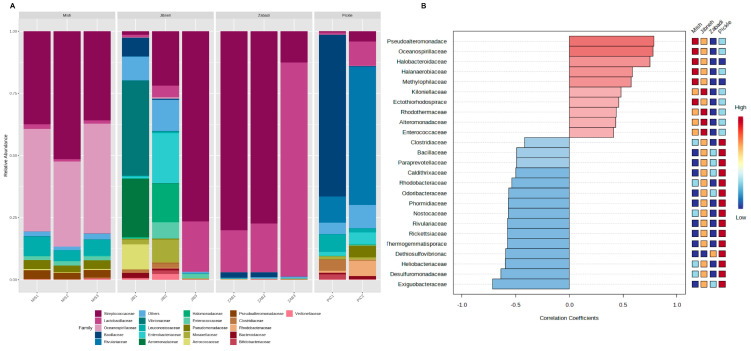
Taxonomic and statistical analysis of bacterial families from 16S amplicon sequencing of dairy and non-dairy fermented foods. (**A**) Relative abundance of dominant bacterial families, and (**B**) Pearson’s correlation of bacterial families with the studied fermented foods.

Families of Streptococcaceae and Oceanospirillaceae were predominantly found in mish samples at relative abundance of >30% (Figure 2A). However, in other dairy and pickle samples, Oceanospirillaceae was detected in <1% of relative abundance, but Streptococcaceae was detected abundantly except in PIC1 (Figure 2A). Lactobacillaceae was abundantly found in zabadi samples (40.9% ± 39.2%) mainly, and a jibneh sample (JIB3 = 20.2%). Families of Vibrionaceae, Aeromonadaceae, and Aerococcaceae were specifically found at >10% relative abundance in JIB1 sample (Figure 2A). Notably, the JIB2 sample showed a higher abundance of Enterobacteriaceae, with a contamination rate of 20.1% (Figure 2A). In the pickle samples, taxa from Bacillaceae were detected at relatively high abundance in PIC1 (65.1%), and Rivulariaceae in PIC2 (55.5%). Pearson’s r correlation analyzed the pattern of top 25 families among the studied fermented foods and identified six families significantly (*p* < 0.05) correlated positively with certain fermented foods (Figure 2B). Xanthobacteraceae, Pseudoalteromonadaceae, Oceanospirillaceae, and Halobacteroidaceae were positively (*p* < 0.05) correlated with mish, followed by jibneh samples, whereas Exiguobacteraceae and Desulfuromonadaceae were correlated positively with pickle and then jibneh (*p* < 0.05) samples compared to mish and zabadi samples (Figure 2B).

Out of the 424 genera found in fermented foods, jibneh gave the biggest number with a corresponding value of 366, followed by pickle with 261, Mish with 208, and zabadi with 142 (Figure S2A). Ninety-two genera were common among the tested fermented foods. The jibneh had the highest number of unique genera, with 82, followed by pickles with 30 (Figure S2A). *Marinomonas* and *Lactococcus* were the dominant genera detected at >30% relative abundance in mish samples, followed by *Leuconostoc*, and these genera were detected at relatively lower abundance in the other fermented foods except the jibneh sample JIB3 that contained 42.6% *Lactococcus* (Figure S2B). *Streptococcus* and *Lactobacillus* were found at relatively high abundance in zabadi and jibneh samples compared to mish and pickle samples. In sample JIB1, *Vibrio* and *Oceanisphaera* were present at a relative abundance of more than 20%, while the *Chromohalobacter* genus was found in high levels only in sample JIB2. Compared with the dairy fermented foods, the dominant genera were different in pickle samples that mainly comprised of *Bacillus*, *Calothrix*, and *Weissella* in PIC1, and *Calothrix*, *Lactobacillus*, and *Phaeobacter* in PIC2 (Figure S2B). Additional variations were noticed in the relatively abundant genera within the jibneh and pickle samples compared to mish and zabadi samples (Figure S2B).

**Figure 3:**
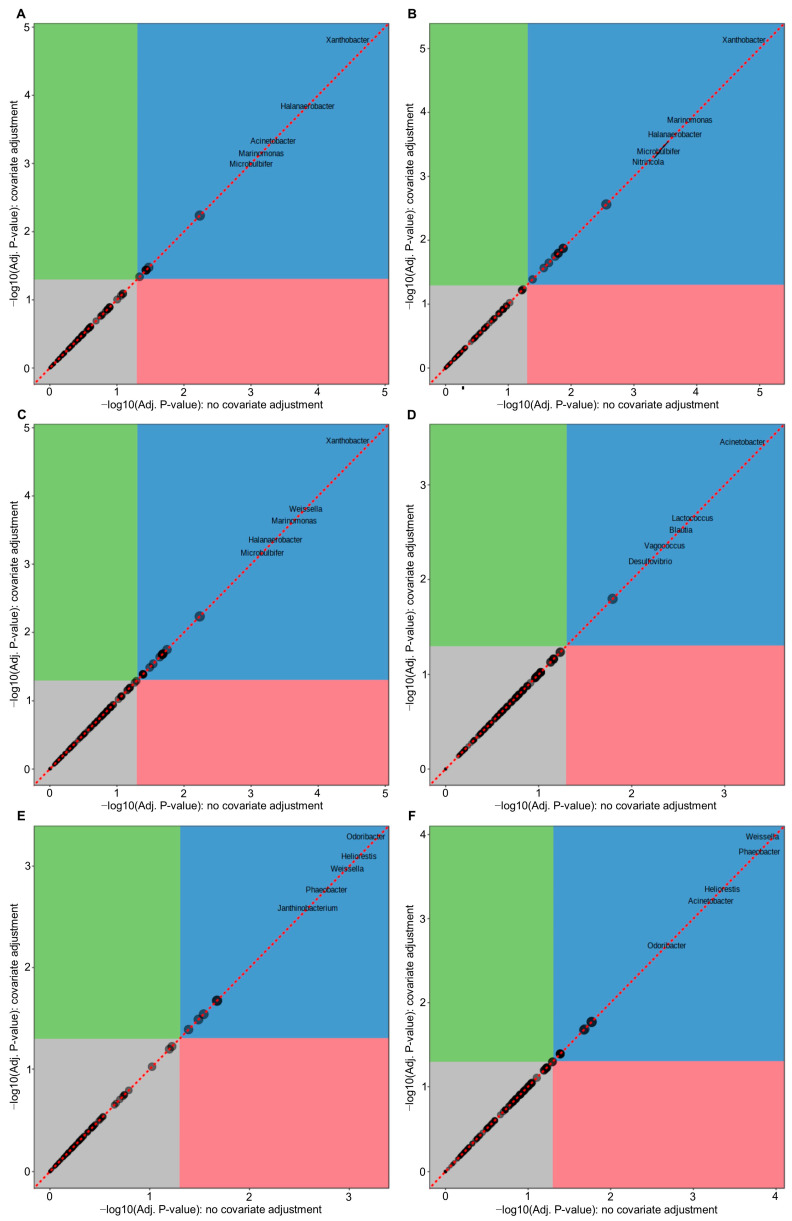
Multi-factor analysis of genera identified from 16S amplicon sequencing among fermented foods. Pairwise comparison of studied fermented foods by microbiome multivariable associations with linear models (MaAsLin2); (**A**) mish vs. jibneh, (**B**) mish vs. zabadi, (**C**) mish vs. pickle, (**D**) jibneh vs. zabadi, (**E**) jibneh vs. pickle, and (**F**) zabadi vs. pickle.

According to MaAsLin 2 (Microbiome Multivariable Associations with Linear Models) analysis, there were significant differences in six genera abundance for the samples between mish and jibneh, nine genera between mish and zabadi, 15 genera between mish and pickle, two genera between jibneh and zabadi, five genera between jibneh and pickle, and nine genera between zabadi and pickle. Further comparative analysis revealed that these were 21 genera significantly different (FDR < 0.05) at least in one fermented food compared to other tested fermented foods (Figure 3). In mish samples, *Xanthobacter*, *Halanaerobacter*, *Marinomonas*, and *Nitrincola* were found to be significantly (FDR < 0.05) different compared to the other tested fermented foods (Figure 3). It was found that *Acinetobacter* was significantly different in jibneh compared to mish and zabadi, as well as in pickle compared to mish and zabadi (FDR < 0.05). However, there was no significant difference between jibneh and pickle samples. The result for the significantly different genera in comparison between fermented food are summarized in Figure 3.

### 3.2. Taxonomy of Shotgun Metagenomic Sequencing

The sequence reads from shotgun sequencing were primarily classified into bacteria (>95%) except in zabadi (82.1%). Fungi and archaea were detected at ≤1.0% abundance, while 17.8% of sequence reads in zabadi were classified as viruses. From the taxonomy at the species level, 367 bacterial species were found in the fermented foods metagenomes. The species of *Lactobacillus helveticus*, *Lactiplantibacillus plantarum*, *Vibrio parahaemolyticus*, *Lactobacillus delbrueckii*, and *Limosilactobacillus fermentum* were common among the tested fermented foods. An increased number of species were identified in the traditional fermented milk mish (320), and less than 50 species were identified among the other fermented foods. In the mish metagenome, there was a relatively high abundance of *Pseudoalteromonas translucida* at 11.8% and *Pseudoalteromonas nigrifaciens* at 8.1% (Figure S3). These species were not found in any other fermented foods. The zabadi sample had a high abundance of *Streptococcus thermophiles*, totaling 59.1%. The jibneh sample contained mostly *Lactococcus lactis* (59.7%) and *Lactobacillus helveticus* (28.3%). The pickle sample had a higher abundance of *Lactobacillus acetotolerans* (80.5%), which is known for being acetic acid tolerant (Figure S3).

Based on probiotic screening, 14 probiotic bacteria were identified in the fermented foods (Table S1). The relative abundance of dominant probiotic bacteria varied among the fermented foods. Jibneh and zabadi were found to contain a high abundance of known probiotics, making up 88.7% and 71.9% of the overall bacterial sequence reads, respectively. The jibneh was mainly composed of *Lactococcus lactis*, while *Streptococcus thermophiles* were predominantly found in zabadi (Table S1). These two probiotic species were present in 3.1% and 2.8% abundance in mish samples, respectively. The known probiotics were detected at a relatively lower abundance of 7.4% and 1.6% in mish and pickle samples, respectively. Collectively, 29 pathogenic, opportunistic, or rare pathogenic bacteria (PORPB) were found in the studied samples (Table S2). The highest quantity of PORPB was observed in mish, which was 23, zabadi had 10, jibneh had 9, while pickle had only 2. The identified PORPB consisted of several clinically relevant pathogens such as *Enterococcus faecalis*, *Enterococcus faecium*, *Escherichia coli*, *Klebsiella pneumoniae*, *Clostridium botulinum*, *Pseudomonas aeruginosa*, and *Vibrio cholera*. Notably, fermented foods metagenomes showed pathogenic bacteria at a very low abundance of less than 0.1% (Table S2).

### 3.3. Binning and Genome Construction

**Figure 4:**
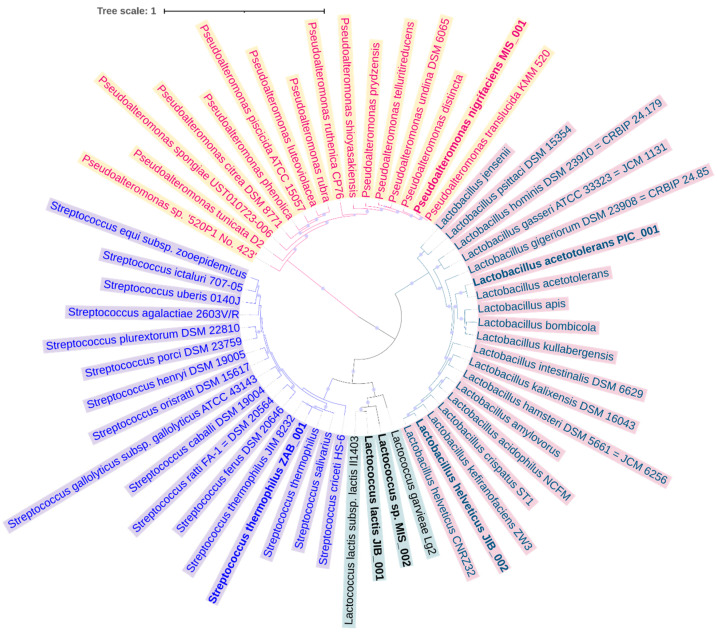
Maximum likelihood phylogenetic analysis of metagenome-assembled genomes retrieved from dairy and non-dairy fermented foods with closely related genomes of bacterial species retrieved from GenBank. Bold font highlights the genomes from this study.

Out of the six MAGs obtained from the reads, five had genomes that were more than 90% complete and had less than 20% contamination (Table S3), and a medium size MAG of MIS_002 was classified as *Lactococcus* species. Phylogenetic analysis and FastANI revealed linkages of the two MAGs from jibneh with *Lactococcus lactis* and *Lactobacillus helveticus* (Figure 4). The MAG of MIS_001 recovered from mish was classified as *Pseudoalteromonas nigrifaciens* and clustered with *Pseudoalteromonas translucida* in the phylogenetic tree (Figure 4). The MAG from zabadi (ZAB_001) was clustered with *Streptococcus thermophiles* and MAG from pickle (PIC_001) was classified as *Lactobacillus acetotolerans*. RAST analysis revealed a high relative abundance of genes associated with carbohydrate metabolism in the MAGs recovered from the studied fermented foods. All genes associated with carbohydrates were categorized into nine sublevels. The highest abundance of genes was associated with di- and oligosaccharides, monosaccharides, C-1 compound metabolism, and fermentation (Table S3). The fermentation genes were mainly classified as mixed acid fermentation and lactate fermentation. Co-factors and vitamin synthesis genes were also detected, including those associated with biotin, thiamin biosynthesis, folate and pterines metabolism, coenzymes NAD and NADP, and riboflavin (Table S3). Resistance genes to daptomycin, triclosan, mupirocin, and fusidic acid were commonly found in most of the identified MAGs. Moreover, multidrug resistance genes and efflux pumps were detected in the MAGs along with metal resistance against arsenic, and copper homeostasis and tolerance (Table S3).

### 3.4. ARGs and Mobile Genetic Elements

**Figure 5:**
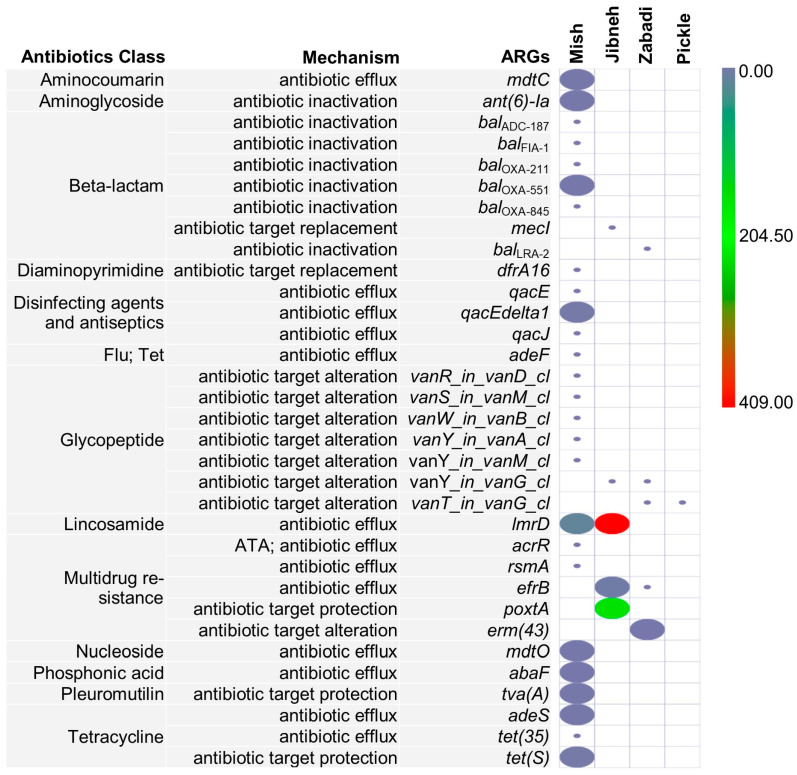
Antimicrobial resistance genes (ARGs) identified from shotgun sequencing of dairy and non-dairy fermented foods. ARG counts normalized to reads per million. Flu, fluoroquinolone; Tet, tetracycline; ATA, antibiotic target alteration.

In the fermented food samples, 33 ARGs were identified produce resistance against ten classes of antibiotics, in addition to multidrug resistance, disinfecting agents, and antiseptics (Figure 5). The majority of the 26 ARGs were identified from mish sample (Figure 5). The resistance genes from mish were linked to produce resistance against lincosamide (*lmrD*), tetracycline (*tet*S, *adeS*), pleuromutilin (*tvaA*), aminoglycoside (*ANT(6)-Ia*), and beta-lactam (*bla*_OXA-551_) antibiotics. The *lmrD* gene, which confers resistance to lincosamide antibiotics, was detected in high abundance in jibneh (409 rpm) sample. Jibneh also contained other resistance genes, such as *poxtA* and *efrB*, leading to multidrug resistance. Five resistance genes were found in the zabadi. These included glycopeptide antibiotic resistance genes from the van cluster (*van*Y, *vanT*), and *bla*_LRA-2_ gene, which produces beta-lactam antibiotic resistance (Figure 5). For pickle, only *vanT* gene in *vanG* cluster was detected. Based on the distinct ARGs, five types of antimicrobial resistance mechanisms were determined in the fermented food metagenomes. These mechanisms primarily involve producing resistance through antibiotic efflux, altering the antibiotic target, and inactivating antibiotics (Figure 5).

The potential for horizontal transfer of ARGs among the studied fermented foods microbiota was determined by screening mobile genetic elements (MGEs). In total, 28 MGEs were identified in the metagenome assemblies, mainly comprised of insertion sequence (IS) (Table S4). A maximum of 18 MGE were found in jibneh, and 12 IS elements were detected in mish samples. The identified IS elements were mostly specific to each sample microbiota, and only *IS*1068 was commonly found in mish, zabadi, and jibneh (Table S4). The *clpL* gene was detected in a mish sample on a contig carrying *IS*30, which conferred resistance to heat and disinfectants and was previously detected in *Lactobacillus plantarum*.

### 3.5. Functional Analysis of Fermented Foods Metagenomes

**Figure 6:**
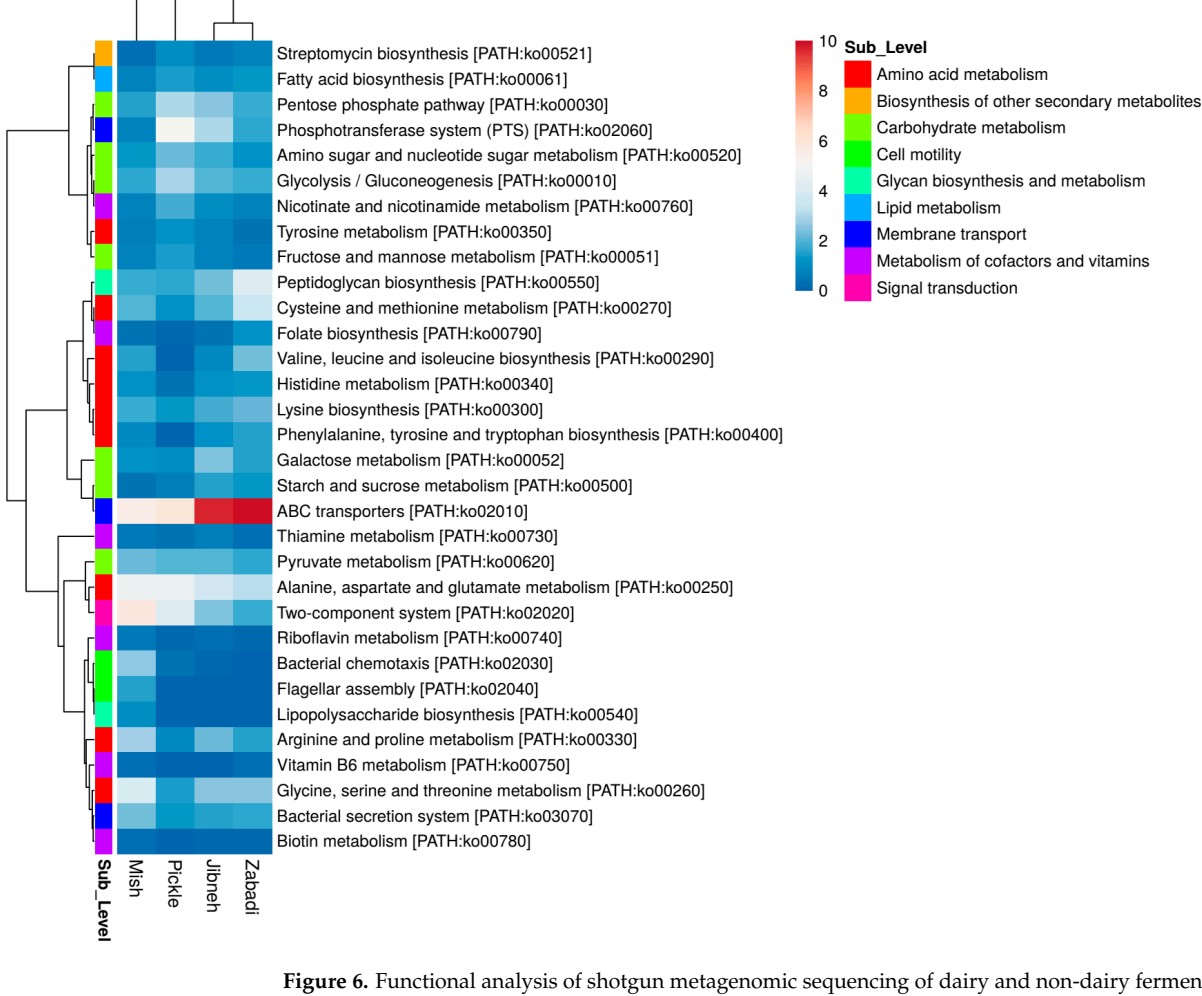
Functional analysis of shotgun metagenomic sequencing of dairy and non-dairy fermented foods. Percentage distribution of metabolic pathways identified from open reading frames mapped to KO (KEGG Orthology) databases. KEGG, Kyoto Encyclopedia of Genes and Genomes.

In the functional analysis using KEGG Orthology database, the metabolism-associated genes were predominantly found (53.1% ± 5.2%) in the fermented foods metagenomes, followed by genetic information processing (24.9% ± 5.9%), and environmental information processing (16.7% ± 1.1%). A relatively high abundance was detected at the KEGG pathways sublevel (level-2) of amino acid metabolism (17.8% ± 4.9%), carbohydrate metabolism (12.7% ± 2.0%), membrane transport (12.2% ± 2.5%), translation, replication and repair (9.0% ± 2.5%), nucleotide metabolism (5.8% ± 1.1%), and metabolism of cofactors and vitamins (5.1% ± 1.2%). At the subcategory level-3, the majority of genes were from the membrane transport category and were associated with ABC transporters [PATH:ko02010], bacterial secretion system [PATH:ko03070], and phosphotransferase system [PATH:ko02060] in the studied metagenomes (Figure 6). The carbohydrate metabolism genes were found at relatively high abundance in jibneh, followed by pickle, mish, and zabadi. During the comparative analysis of the functional potential, pentose phosphate pathway [PATH:ko00030], amino sugar and nucleotide sugar metabolism [PATH:ko00520], and fructose and mannose metabolism [PATH:ko00051] were found at relatively high abundance in pickle compared to jibneh sample and detected at <1.5% in other tested fermented foods. Glycolysis/gluconeogenesis [PATH:ko00010], and pyruvate metabolism [PATH:ko00620] were the other carbohydrate metabolism pathways commonly found at >1.0% relative abundance in the studied metagenomes (Figure 6).

Relative to pickle, a higher abundance of genes responsible for the glycine, serine, and threonine [PATH:ko00260], arginine and proline [PATH:ko00330], cysteine and methionine [PATH:ko00270], lysine biosynthesis [PATH:ko00300], histidine [PATH:ko00340], phenylalanine, tyrosine and tryptophan biosynthesis [PATH:ko00400], and valine, leucine and isoleucine biosynthesis [PATH:ko00290] were detected in the dairy fermented food samples (Figure 6). However, alanine, aspartate and glutamate [PATH:ko00250], and tyrosine metabolism [PATH:ko00350] showed higher abundance in pickle metagenome. Among the cofactors and vitamin synthesis, the genes responsible for nicotinate and nicotinamide metabolism [PATH:ko00760] were found at relatively high abundance in pickle compared to dairy fermented foods. The pathways for biotin metabolism [PATH:ko00780] and lipoic acid metabolism [PATH:ko00785] were not detected in pickle metagenome (Figure 6). In the dairy fermented foods, pathways of ubiquinone and other terpenoid-quinone biosynthesis [PATH:ko00130], folate biosynthesis [PATH:ko00790], and vitamin B6 metabolism [PATH:ko00750] were detected at relatively high abundance compared to pickle. Moreover, pathways of secondary metabolites, streptomycin biosynthesis [PATH:ko00521] were found at relatively high abundance in pickle (1.0%) and was present <1.0% in other samples (Figure 6). The penicillin and cephalosporin biosynthesis [PATH:ko00311] pathway was detected specifically in jibneh and mish metagenomes. The *damX* gene for an inner membrane protein involved in bile resistance, and *mdt*ABCD (multidrug resistance protein) were found in mish. The enzymes involved in horizontal gene transfer, including integrase, DNA primase, and phage lysin, were detected in all the fermented foods metagenomes.

## 4. Discussion

In this study, a combinatorial sequencing approach of 16S amplicon sequencing and shotgun sequencing was employed to study the microbial diversity and the functional potential of the traditional Arabian fermented milk foods and non-dairy fermented foods commonly consumed in the Arabian Peninsula. We identified microbial diversity, probiotics, and variations in relatively dominant bacterial taxa in the studied fermented foods metagenomes. Consistent with previous studies for fermented foods, Firmicutes, Proteobacteria, Bacteroidetes, and Actinobacteria were commonly found in the studied samples [33,34,35]. The traditional Arabian mish samples contained a high abundance of both Firmicutes and Proteobacteria. Notably, the *Marinomonas* genus from Gammaproteobacteria and *Lactococcus* and *Leuconostoc* from Firmicutes were predominantly found in mish samples. These genera were found in lower abundance in most of the other fermented foods that were examined. Limited studies have evaluated the microbiology of mish, and the available culture analysis reported *Staphylococcus aureus*, psychotropic bacteria, yeast, and lactobacilli from mish samples mainly conducted in Sudan [36,37]. Abdalla and Zubeir identified pathogenic bacteria of *E. coli*, *S. aureus*, *Streptococcus* spp., and *Salmonella* spp. in a study of characterizing microbial hazards associated with mish and other fermented milk [38].

Taxa of *Streptococcus* and *Lactobacillus* were found in relatively high abundance in zabadi and jibneh samples. However, genera of *Bacillus*, *Calothrix*, *Weissella*, and *Lactobacillus* were abundantly found in pickle samples indicates variation in bacterial community composition among different fermented food types as observed in previous studies and could be linked to the fermented foods substrates, production recipes, environment influence, and manufacturing conditions [2,6,7]. A recent study from Greece identified lactic acid bacteria, followed by yeasts, Enterobacteria, pseudomonads, and staphylococci in the cheese samples using culture-dependent and 16S rRNA gene sequence analysis [39]. *Bacillus*, *Lactobacillus*, *Clostridium*, and *Streptococcus* were the most prevalent taxa in pickles, and our findings were consistent with the studies from fermented soybeans and vegetables [40,41]. Overall, in accordance with taxonomic analysis, six MAGs were retrieved from the tested fermented foods metagenomes classified as *Lactococcus lactis* and *Lactobacillus helveticus* from jibneh, *Streptococcus thermophiles* from zabadi, and *Lactobacillus acetotolerans* were identified from the pickle sample. However, *Pseudoalteromonas nigrifaciens* genome was retrieved from mish along with *Lactococcus* species. The source of *Pseudoalteromonas nigrifaciens* in mish sample is not known, but a previous study by Kothe et al. retrieved *P. nigrifaciens* from the cheese sample using a metagenomic approach [42]. Furthermore, a large sample size would be necessary to fully comprehend the observed intra-sample variation in bacterial community composition among the fermented foods. It is important to acknowledge that the current study is limited by its relatively small sample size.

The probiotic species reported in dairy fermented foods were different relative to the beneficial taxa of non-dairy fermented food, probably due to the nature of food substrates. In this study, the enhanced level of *Streptococcus thermophilus* and *Lactococcus lactis* were identified in dairy fermented foods, mainly in zabadi (59.1%) and jibneh (59.7%), respectively. *Streptococcus thermophiles* and *Lactococcus lactis* are the prominent probiotics in the dairy industry and possess health beneficial immunomodulatory ability [43,44,45]. The *Lactobacilli* strains were also reported in traditional Polish vegetable fermented food [46]; in line with this study, the enhanced abundance of Lactobacillales was found in pickle metagenome. Overall, an increased number of known probiotic species were found in jibneh (*n* = 11) followed by in mish (*n* = 8) samples. The safety of food is a major concern for consumers. Several PORPB were found in the studied samples, mainly in mish (*n* = 23) metagenome, but the relative abundance of those clinically important pathogenic bacteria was less than 0.1% as previously reported [2,7]. Two pathogenic bacteria of *Vibrio parahaemolyticus* (0.06%) and *Clostridium botulinum* (0.01%) were detected in pickle metagenome. Similarly, an increased number of ARGs were found in mish (26 ARGs) metagenome conferring resistance to lincosamide, tetracycline, pleuromutilin, aminoglycoside, glycopeptide, and beta-lactam antibiotics. Genes causing glycopeptide, beta-lactam antibiotic, and multidrug resistance were also found in the jibneh and zabadi metagenomes. ARGs have been found in fermented vegetables and dairy products at varying levels, reducing the effectiveness of clinically important antibiotics such as aminoglycosides, fluoroquinolones, beta-lactams, tetracycline, lincosamides, fosfomycin, nitrofurans, nitroimidazoles, phenicols, macrolides, and rifamycin [6,47,48]. As a result of food-borne ARGs entering the body, the gut microbiome can absorb them and cause adverse health effects [49].

Humans fail to synthesize several metabolites, including SCFAs and vitamins, whereas an exogenous supply is needed for the overall development [18,50,51]. Microbes residing in the large intestine rely on the host’s undigested leftover dietary substrates, which undergo saccharolytic bacterial fermentation to produce the bacterial metabolites, mainly SCFAs (acetate, butyrate, and propionate) [52]. In this study, functional analysis of the fermented food metagenomes and MAGs highlight the metabolic pathways of carbohydrate metabolism, and vitamins biosynthesis including those associated with biotin, riboflavin, thiamin biosynthesis, folate, and pterines metabolism. Other studies have also reported the abundance of probiotic bacteria such as *Lactobacilli* species in dairy and non-dairy fermented foods and encoding the genes for vitamin biosynthesis, fermentation, and secondary metabolites production [2,7,53]. Vitamins are the necessary micronutrient that serves as the precursor molecules involved in the biosynthesis of enzymes that participate in the biochemical reactions of the cells [18]. Moreover, fermented food could enrich bacteria such as *Propionibacterium freudenreichii*, *Bifidobacteria*, and *Lactobacilli* in the gut that could synthesize most of the water-soluble vitamins, including biotin, cobalamin, riboflavin, folates, pyridoxine and thiamine, thus able to fulfill the requirement of the vitamins for the host [17,18,50,54,55,56]. Similarly, biotin was commonly found in the MAGs retrieved from the studied dairy fermented is a cofactor of many enzymes that participate in gluconeogenesis, amino acid, and fatty acid biosynthesis [57]. Cofactors and vitamin metabolism pathways were found at relatively high abundance in dairy fermented foods, mainly in mish (6.5%) followed by zabadi (5.4%) compared to pickle (3.7%). Similarly, amino acid metabolism pathways were found at relatively high abundance in mish (23.0%), followed by zabadi (19.5%) and jibneh (17.1%) compared to pickle (11.4%). A high abundance of asparagine synthetase was found in pickle and jibneh metagenomes. The role of asparagine synthetase expression was reportedly crucial in healthy neuronal development [58]. Overall, microbiological analysis has uncovered the nutritional function of the studied fermented foods metagenomes that could beneficially affect consumer health and gut microbiota.

## 5. Conclusions

This study provided an integrated analysis of dairy and non-dairy fermented foods in the Arabian Peninsula, linking microbial diversity and functional gene content. Noticeably distinct bacterial diversity was observed among dominant taxa at lower taxonomic levels in traditional fermented milk mish, when compared to other dairy and pickle samples. Genera such as *Marinomonas*, *Lactococcus*, and *Leuconostoc* were predominantly identified in mish samples, whereas their presence was relatively less pronounced in most other studied fermented food samples. *Streptococcus* and *Lactobacillus* were found in relatively high abundance within zabadi and jibneh samples. The mish metagenome exhibited an increased number of ARGs and pathogenic bacteria; however, these were in minor relative abundance. Furthermore, the outcomes of this study suggest that Arabian fermented foods serve as significant sources of probiotic bacteria, fostering carbohydrate and amino acid metabolism, as well as vitamin biosynthesis. Presently, the overall risk to human health stemming from the consumption of these foods is assessed to be low. Nevertheless, responsible production practices and ongoing research are imperative to oversee the safety, nutritional advantages, and potential implications for public health associated with these fermented foods.

## Data Availability

Sequencing data were deposited into the European Nucleotide Archive (ENA) under project no. PRJEB19968 and sample accession numbers; MIS1, SAMEA103945074; MIS2, SAMEA103945075; MIS3, SAMEA103945076; Mish, SAMEA104419883; JIB1, SAMEA103945069; JIB2, SAMEA103945070; JIB3, SAMEA103945068; Jibneh, SAMEA104419881; ZAB1, SAMEA103945062; ZAB2, SAMEA103945063; ZAB3, SAMEA103945064; Zabadi, SAMEA104419885; PIC1, SAMEA103945073; PIC2, SAMEA103945072; Pickle, SAMEA104419884.

## Supplementary Materials

The following supporting information can be downloaded at: https://www.mdpi.com/article/10.3390/foods12183342/s1, Figure S1: Relative abundance of dominant bacterial phyla identified from 16S amplicon sequencing of dairy and non-dairy fermented foods. Figure S2: Analysis of bacterial communities at genus taxonomic level from 16S amplicon sequencing. (A) Venn diagrams for the common and unique genera among the dairy and non-dairy fermented foods. (B) Relatively abundance of top 20 bacterial genera identified from 16S amplicon sequencing. Figure S3: Taxonomic analysis of bacterial species identified at ≥1% relative abundance in at least one fermented food shotgun sequencing. Table S1: Percentage relative abundance of probiotic bacteria found in the taxonomic analysis of studied fermented foods shotgun sequencing. Table S2: Percentage relative abundance of pathogenic, opportunistic, and rare human pathogenic bacteria found in the taxonomic analysis of shotgun sequencing data from the studied fermented foods. Table S3: Genomic properties and annotation of the metagenome-assembled genomes from the shotgun sequencing of dairy and non-dairy fermented foods. Table S4: Mobile genetic elements identified in metagenomic assemblies from the studied fermented foods.
