# Supplementary material for: Metagenomic Insights into the Microbiome and Resistance Genes of Traditional Fermented Foods in Arabia
Source: Foods. 2023 Sep 6;12(18):3342. doi: 10.3390/foods12183342 (PMC10528461; doi:10.3390/foods12183342)
Supplement: Supplementary file 1 [file foods-12-03342-s001.zip › foods-2584470-supplementary.pdf]

Supplementary Data

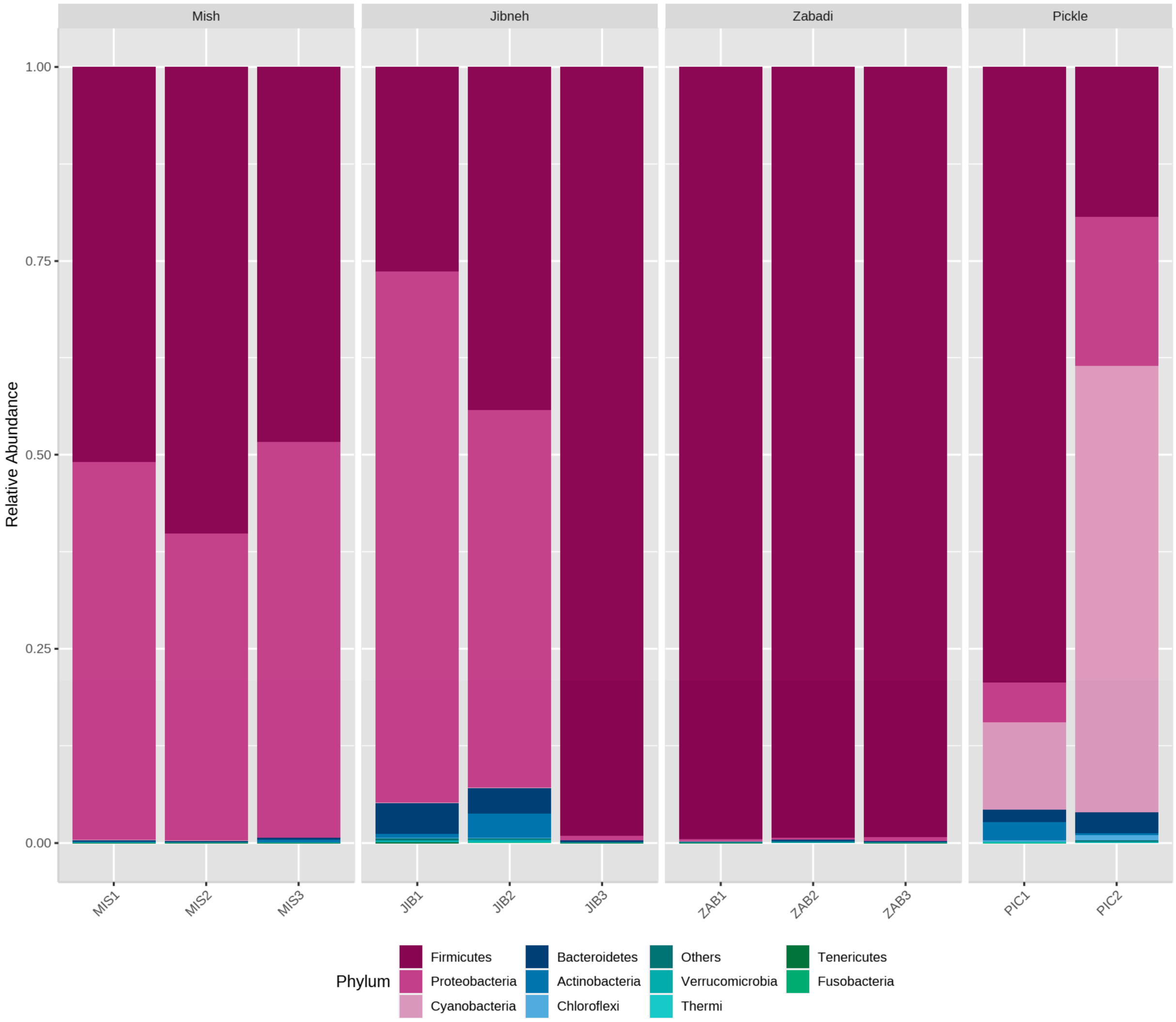

Figure S1. Relative abundance of dominant bacterial phyla identified from 16S amplicon sequencing of dairy and non-dairy fermented foods.

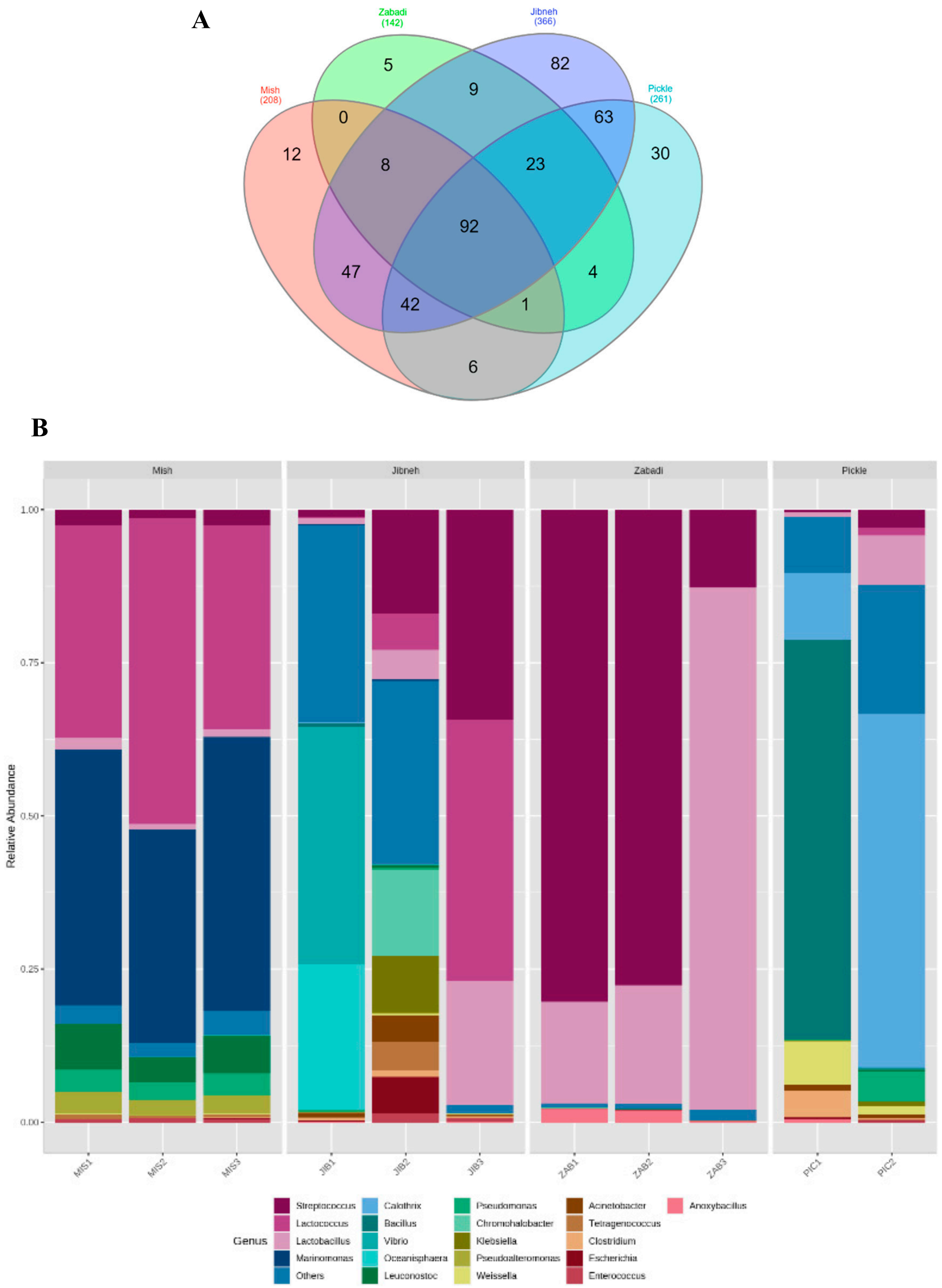

Figure S2. Analysis of bacterial communities at genus taxonomic level from 16S amplicon sequencing. (A) Venn diagrams for the common and unique genera among the dairy and non-dairy fermented foods. (B) Relatively abundance of top 20 bacterial genera identified from 16S amplicon sequencing**.**

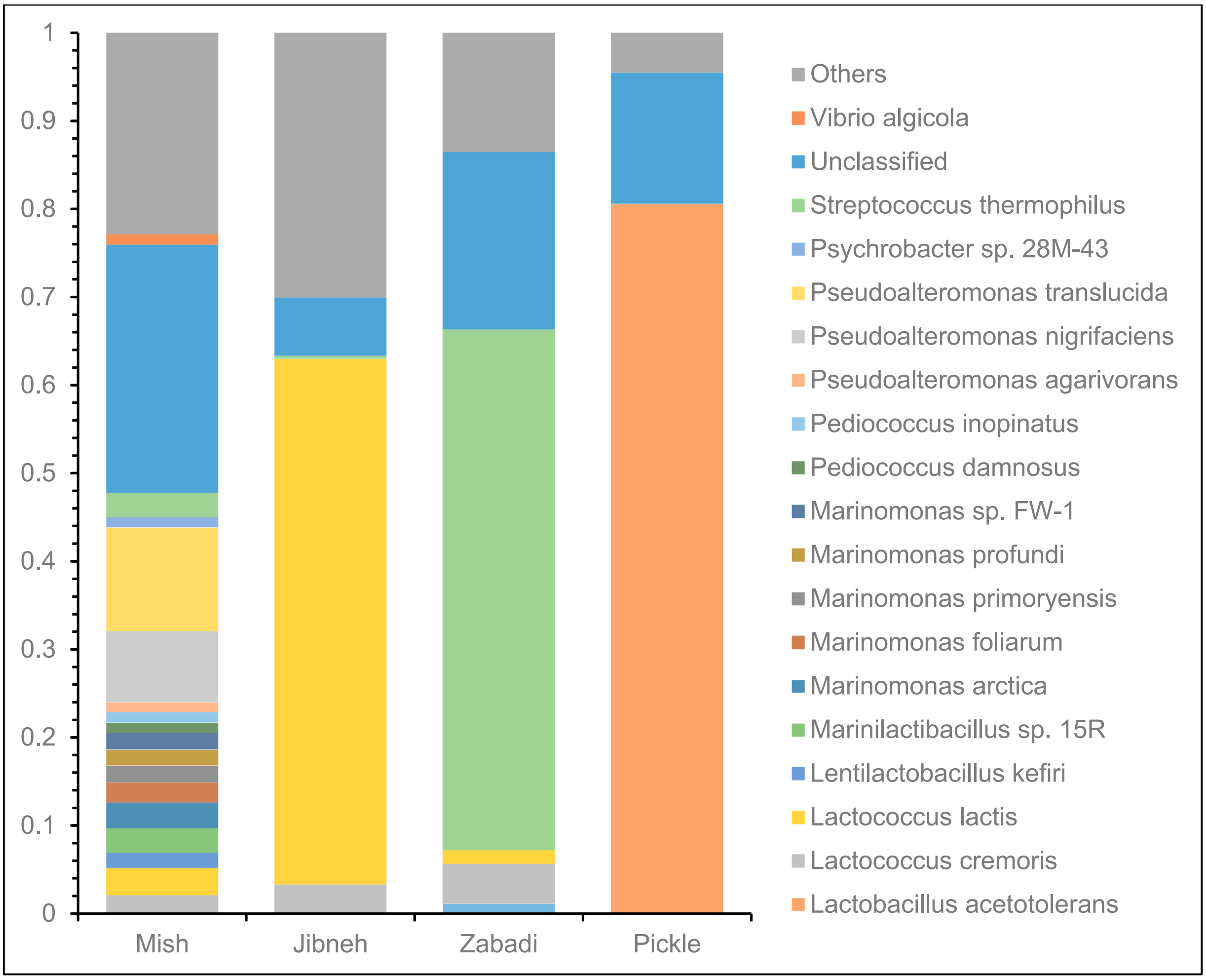

Figure S3. Taxonomic analysis of bacterial species identified at ≥1% relative abundance in at least one fermented food shotgun sequencing.

Table S1. Percentage relative abundance of probiotic bacteria found in the taxonomic analysis of studied fermented foods shotgun sequencing.

**Species**	**Mish (%)**	**Jibneh (%)**	**Zabadi (%)**	**Pickle (%)**
Streptococcus thermophilus	2.76	0.35	59.09	0.00
Streptococcus salivarius	0.07	0.01	0.51	0.00
Pediococcus acidilactici	0.00	0.01	0.00	0.02
Leuconostoc mesenteroides	0.20	0.00	0.00	0.00
Lactococcus lactis	3.08	59.66	1.56	0.00
Lactococcus garvieae	0.16	0.02	3.98	0.00
Lactobacillus johnsonii	0.00	0.05	0.00	0.06
Lactobacillus helveticus	0.08	28.34	0.05	0.87
Lactobacillus gasseri	0.00	0.00	0.00	0.16
Lactobacillus delbrueckii	0.91	0.04	6.62	0.10
Lactobacillus crispatus	0.00	0.10	0.00	0.33
Lactobacillus acidophilus	0.00	0.06	0.00	0.07
Enterococcus lactis	0.00	0.00	0.04	0.00
Enterococcus faecium	0.08	0.03	0.00	0.00

Table S2. Percentage relative abundance of pathogenic, opportunistic, and rare human pathogenic bacteria found in the taxonomic analysis of shotgun sequencing data from the studied fermented foods.

Species	Mish	Jibneh	Zabadi	Pickle
Acinetobacter junii	0.01	0.00	0.00	0.00
Clostridium botulinum	0.03	0.00	0.46	0.01
Enterobacter cloacae	0.02	0.00	0.00	0.00
Enterococcus casseliflavus	0.02	0.00	0.00	0.00
Enterococcus durans	0.00	0.01	0.00	0.00
Enterococcus faecalis	0.05	0.01	0.00	0.00
Enterococcus faecium	0.08	0.03	0.00	0.00
Enterococcus lactis	0.00	0.00	0.04	0.00
Escherichia coli	0.05	0.00	0.00	0.00
Klebsiella pneumoniae	0.08	0.00	0.00	0.00
Lactococcus garvieae	0.16	0.02	3.98	0.00
Leclercia adecarboxylata	0.01	0.00	0.00	0.00
Leuconostoc pseudomesenteroides	0.01	0.01	0.00	0.00
Proteus mirabilis	0.02	0.00	0.00	0.00
Pseudomonas aeruginosa	0.01	0.00	0.00	0.00
Pseudomonas entomophila	0.01	0.00	0.00	0.00
Stenotrophomonas maltophilia	0.01	0.00	0.00	0.00
Streptococcus agalactiae	0.00	0.02	0.04	0.00
Streptococcus ferus	0.01	0.00	0.00	0.00
Streptococcus infantarius	0.02	0.00	0.00	0.00
Streptococcus mitis	0.00	0.00	0.04	0.00
Streptococcus mutans	0.00	0.00	0.05	0.00
Streptococcus oralis	0.00	0.00	0.06	0.00
Streptococcus salivarius	0.07	0.01	0.51	0.00
Streptococcus vestibularis	0.03	0.00	0.42	0.00
Vibrio cholerae	0.07	0.00	0.00	0.00
Vibrio parahaemolyticus	0.23	0.19	0.08	0.06
Vibrio vulnificus	0.02	0.00	0.00	0.00
Weissella cibaria	0.01	0.01	0.00	0.00

Table S3. Genomic properties and annotation of the metagenome-assembled genomes from the shotgun sequencing of dairy and non-dairy fermented foods.

**Features**	MIS_001	**MIS_002**	**JIB_001**	**JIB_002**	**ZAB_001**	**PIC_001**
Classification	*P. nigrifaciens*	*Lactococcus sp.*	*L. lactis*	*L. helveticus*	*S. thermophilus*	*L. acetotolerans*
FastANI ANI	98.56		98.54	98.68	98.65	98.67
Coarse consistency (%)	99.5	85.3	99.5	98.1	99.7	99.3
Fine consistency (%)	97.6	78.5	98.7	94.2	91.5	98.6
Completeness (%)	99.8	45.3	99.2	98.1	100	94.2
Contamination (%)	1.2	14.7	0.8	8.8	17.3	0.7
Contig count	252	1241	149	592	477	116
Contigs N50 (bp)	61120	1407	32159	9319	13829	18281
Contigs L50	24	456	21	76	38	20
Protein-encoding genes with functional assignment	2411	1596	1701	2123	1934	1002
Protein-encoding genes without functional assignment	1689	1353	816	1142	898	408
Protein-encoding feature coverage (%)	96.66	161.36	105.36	121.91	123.57	101.28
Features that are hypothetical (%)	41.2	45.88	32.42	34.98	31.71	28.94
Features that are in local protein families (%)	91.32	31.13	94.2	66.43	75.95	96.31
**Carbohydrates**						
Amino sugars	9	0	4	0	0	0
Central carbohydrate metabolism	0	0	0	0	0	0
Carboxylic acids	0	0	0	0	2	0
CO2 fixation	0	0	1	1	1	0
Di- and oligosaccharides	14	3	11	18	11	0
Fermentation						
Acetoin, butanediol metabolism	0		0	1	6	0
Fermentations: Lactate	0	11	11	14	17	10
Fermentations: Mixed acid	0	15	12	14	23	0
Acetolactate synthase	4	0	0	0	2	0
Monosaccharides	11	1	6	2	1	2
C-1 compound metabolism	3	4	9	11	17	3
Polysaccharides	1	0	6	0	0	0
**Cofactors, Vitamins, Prosthetic Groups, Pigments**						
Tetrapyrroles	31	5	3	1	4	0
Thiamin_biosynthesis	3	5	7	4	5	10
Biotin	40	10	19	14	8	0
Coenzyme A	7	0	3	2	3	2
Folate and pterines	38	0	30	21	35	3
Lipoic acid	19	6	7	0	6	0

NAD and NADP	14	0	7	16	34	18
Pyridoxine	10	6	8	8	8	0
Quinone cofactors	13	0	9	0	0	0
Riboflavin, FMN, FAD	10	0	7	5	4	3
Fe-S clusters	13	0	10	0	0	0
**Resistance to antibiotics and toxic compounds**						
Arsenic resistance	0	0	0	0	3	0
Copper homeostasis: copper tolerance	3	1	1	0	0	0
Resistance to daptomycin	3	11	9	9	9	6
Aminoglycoside modifying enzymes	0	0	0	1	0	0
Resistance to triclosan	1	1	3	1	1	1
Cadmium resistance	0	3	2	2	0	0
Fusidic acid resistance	2	5	2	2	5	0
Mupirocin resistance	1	0	1	1	1	1
Fusaric acid resistance	3	0	0	0	0	0
Polymyxin resistance	1	6	0	0	0	0
Resistance to chromium compounds	1	0	0	0	0	0
Antibiotic efflux	1	1	1	0	0	0
Glycopeptide resistance *vanY*, *vanG*	0	0	1	0	1	1

Values are based on the number of genes detected for the respective functions, except indicated with the (%) symbol.

Table S4. Mobile genetic elements identified in metagenomic assemblies from the studied fermented foods.

Samples	MGE	Synonyms	Identity	Coverage	Substitution
Mish	*ISShfr5*		0.92	1.00	206
	IS5		1.00	1.00	5
	ISLpl1	*ISLp1*	1.00	1.00	5
	ISLla3		1.00	1.00	5
	IS905	*IS905B*; *IS905A*	0.99	1.00	11
	ISLpl1	*ISLp1*	1.00	1.00	5
	IS712		1.00	1.00	0
	ISSth1		1.00	1.00	1
	ISSth1b	*ISSth1*	1.00	1.00	3
	ISPpi1		0.94	1.00	41
	IS1068	*IS904N*; *IS904*	0.99	1.00	5
	ISStrs1		0.92	0.98	48
Zabadi	*ISSth6*		1.00	1.00	0
	ISLll1		0.98	0.99	12
	ISS1X		1.00	1.00	3
	ISL4		0.99	0.99	12
	IS1068	*IS904N*; *IS904*	0.99	1.00	6
Jibneh	*IS712*		1.00	1.00	0
	ISLla2		0.93	0.99	73
	ISL5		0.98	0.99	9
	ISL5		0.98	0.99	9
	IS-LL6		0.98	1.00	19
	ISS1W		1.00	1.00	2
	IS1165		0.98	0.99	22
	ISLjo1		0.95	1.00	51
	ISLhe5		0.99	1.00	10
	ISS1X		1.00	1.00	1
	ISLgar3		0.99	1.00	8
	ISS1N		0.96	0.99	27
	ISLhe9		1.00	1.00	0
	ISLhe61		1.00	1.00	0
	IS905	*IS905B*; *IS905A*	1.00	1.00	3
	IS1068	*IS904N*; *IS904*	0.99	0.98	6
	ISS1N		0.98	1.00	18
	cn_9850_ISL5		0.98	0.99	9
Pickle	*ISLhe7*		0.98	0.99	9

MGE, mobile genetic elements.
